# Supplementary material for: KRAP tethers IP3 receptors to actin and licenses them to evoke cytosolic Ca2+ signals
Source: Nat Commun. 2021 Jul 23;12:4514. doi: 10.1038/s41467-021-24739-9 (PMC8302619; doi:10.1038/s41467-021-24739-9)
Supplement: Supplementary file 1 — Supplementary Information [file 41467_2021_24739_MOESM1_ESM.pdf]

KRAP tethers IP_3 receptors to actin and licenses them to evoke cytosolic Ca^{2+} signals

Nagendra Babu Thillaiappan[1,2,*], Holly A Smith[1], Peace Atakpa-Adaji[1] & Colin W Taylor[1,*]

[1]Department of Pharmacology, Tennis Court Road, Cambridge, CB2 1PD, UK.
[2]Department of Basic Medical Sciences, College of Medicine, QU Health, Qatar University, Doha, Qatar.

Supplementary Figures and Table

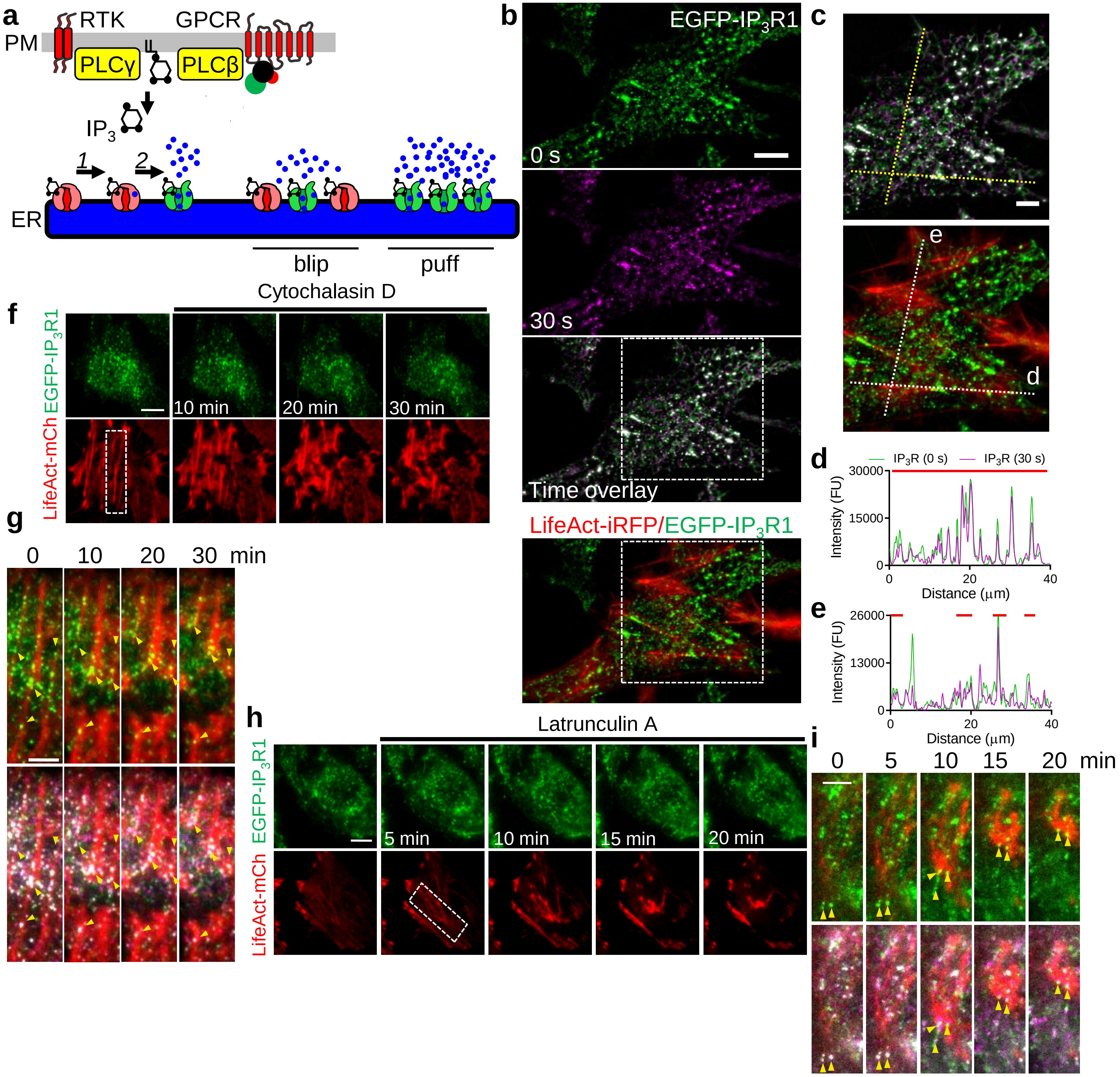

Supplementary Fig. 1 Immobile IP_3Rs associate with actin. a, G protein-coupled receptors (GPCR) and receptor tyrosine kinases (RTK) stimulate phospholipases C (PLC) to produce IP_3. When IP_3 binds to each subunit of a tetrameric IP_3R, the IP_3R is primed to respond to Ca^{2+} (*1*). Ca^{2+} binding then causes pore opening (*2*) and a rapid flow of Ca^{2+} into the cytosol. Opening of a single IP_3R causes a transient local increase in cytosolic [Ca^{2+}], a 'Ca^{2+} blip'. Regulation of IP_3Rs by IP_3 and Ca^{2+} allows IP_3Rs to mediate regenerative Ca^{2+} signals. The smallest of these regenerative signals is a Ca^{2+} puff, a brief (~100 ms) local increase in cytosolic [Ca^{2+}] arising from coordinated opening of a few clustered IP_3Rs as Ca^{2+} released by an active IP_3R ignites the activity of its neighbours. Whether evoked by receptor activation or photolysis of ci-IP_3, most Ca^{2+} puffs occur at fixed sites close to the PM. **b**,**c**, TIRF images of a cell expressing LifeAct-iRFP670 (actin) show EGFP-IP_3R1 (green and magenta at 0 s and 30 s) and their overlay (immobile IP_3R appear white); and actin filaments (red) overlaid with EGFP-IP_3R1. Boxed areas enlarged in c. Scale bars,10 μm (b) and 5 μm (c). Images typical of 7 cells from 5 independent dishes. **d**,**e**, Transects (dashed lines in c) show EGFP-IP_3R1 fluorescence intensity (at 0 s and 30 s) along an actin filament (d) or perpendicular to it (e). Red lines indicate regions with actin. FU, fluorescence unit. Summary in **Fig. 1d**. **f-i**, TIRF images show EGFP-IP_3R1 and actin (LifeAct-mCherry) after treatment with cytochalasin D (10 μM, f,g) or latrunculin A (5 μM, h,i) to depolymerize actin filaments. Enlargements of boxed regions (g,i) show overlays of actin with EGFP-IP_3Rs (top) or with immobile IP_3Rs (white, determined by overlaying EGFP images collected at 0 and 20 s). Despite loss of most actin filaments, many 'immobile' IP_3Rs (arrows) remain associated with short residual filaments. Scale bars,10 μm (f,h) or 5 μm (g,i). Results are typical of at least 5 analyses. Changes in cell shape during actin depolymerization prevent direct comparison of specific IP_3R puncta before and after treatment, but it does not preclude identification of immobile puncta, which requires comparison of two images 20 s apart. See also **Supplementary Movies 1-3**.

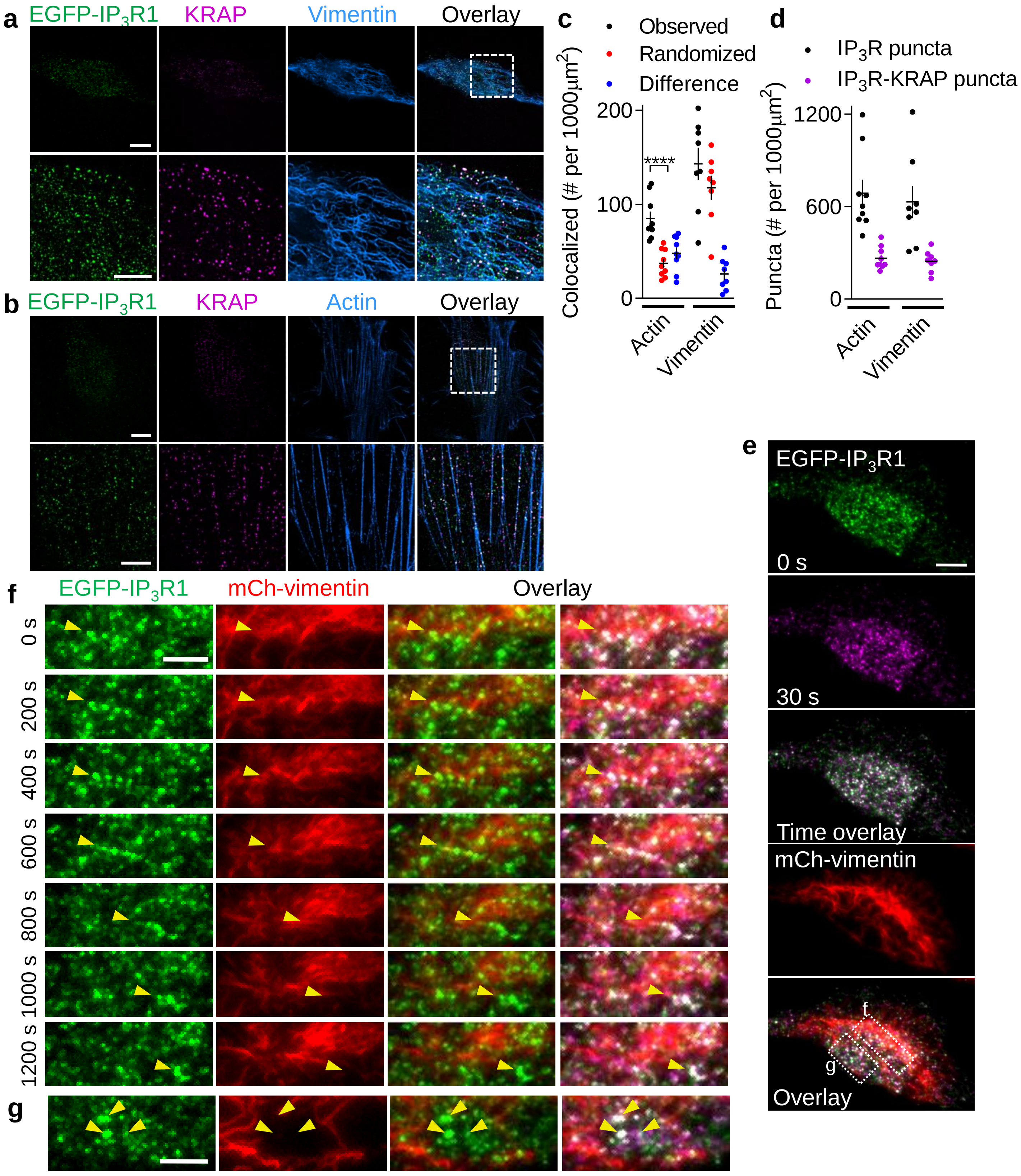

Supplementary Fig. 2 Immobile IP$_3$Rs are not associated with intermediate filaments.
Legend on next page.

Supplementary Fig. 2 Immobile IP_3Rs are not associated with intermediate filaments.
Figure on preceding page.

a,**b**, Superresolution confocal images (single plane, close to PM) show EGFP-IP_3R1 HeLa cells immunostained for EGFP (GFP-Booster), KRAP and either vimentin (a) or actin (b). Results typical of many cells from 2 independent experiments. Scale bar, 10 μm (5 μm in enlargements). **c**, Similar images were used to determine numbers of puncta wherein KRAP and IP_3R were colocalized (centroid separations ≤ 2 pixels, ie ≤130 nm) and their colocalization with actin or vimentin. For these analyses, regions of interest (ROI, 150-570 pixels, typically 30% of cell area) were selected where actin or vimentin staining was not so dense as to preclude meaningful colocalization analyses. To determine the statistical significance of colocalization with actin (or vimentin), the ROI was cut into 66-198 rectangular blocks (30 x 30 pixels) containing the actin (or vimentin) mask. The fragments were then randomly distributed onto the original ROI with its images of KRAP and IP_3R. Colocalization of the unchanged KRAP-IP_3R puncta with the randomized actin (or vimentin) mask was then determined. Because vimentin staining is more widespread than actin staining, the ROIs amenable to analyses were smaller for vimentin (336 ± 72 μm^2) than for actin (482 ± 153 μm^2). Numbers of KRAP-IP_3R puncta colocalized with actin or vimentin are therefore expressed relative to the area analysed (# per 1000 μm^2). Mean ± s.e.m., n = 9 (actin) or 8 cells (vimentin). $^{****}P < 0.0001$, Student's *t*-test. **d**, Numbers of IP_3R puncta and colocalized KRAP-IP_3R puncta in the cells used for the analyses in c. Mean ± s.e.m., n = 9 (actin) or 8 cells (vimentin). **e**, TIRF images, typical of 3 analyses, of EGFP-IP_3R1 HeLa cells expressing mCherry-vimentin show effects of latrunculin A (5 μM, to depolymerize actin) on the distribution of immobile IP_3Rs. Images of EGFP-IP_3R are shown at 30-s intervals (green and magenta at 0 and 30 s respectively, such that immobile IP_3R puncta appear white in the time overlay). Scale bar, 10 μm. **f**,**g**, Enlargements of boxed areas show, at the indicated times after addition of latrunculin A (left to right): EGFP-IP_3R, mCh-vimentin, overlay of EGFP-IP_3R and mCh-vimentin, and mobile (green or magenta) and immobile IP_3R puncta (white) overlaid on mCh-vimentin. Scale bars, 5 μm. Arrows highlight strings of immobile IP_3R puncta that are either not associated with vimentin (g), or as actin depolymerizes retreat to regions devoid of vimentin (f). Images typical of 3 cells from 3 independent dishes. Perturbation of vimentin filaments by depolymerization of actin is unsurprising since intermediate filaments (IF) and actin filaments interact, and vimentin associates with actin[30-32]. We attempted to disrupt vimentin filaments using withaferin A[30] (250 nM - 2 μM, 4 hr), but we failed to find treatments that effectively disrupted IF without perturbing actin filaments. Others have reported similar effects[30]. The results demonstrate that although immobile IP_3R puncta are tightly associated with actin (**Fig. 1, Supplementary Fig. 1, Supplementary Movies 1-3**), they do not associate specifically with IF.

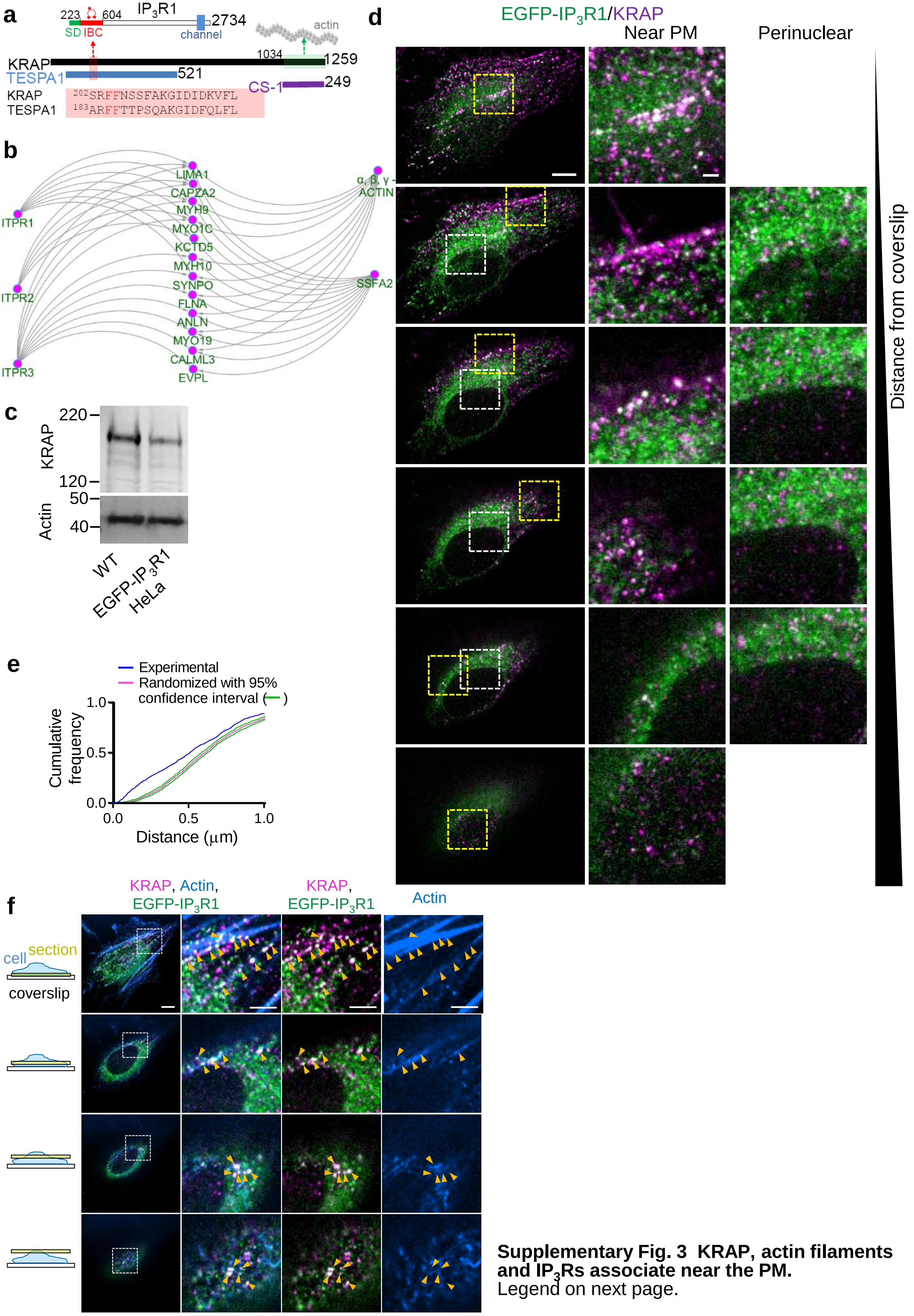

Supplementary Fig. 3 KRAP, actin filaments and IP$_3$Rs associate near the PM.
Legend on next page.

Supplementary Fig. 3 KRAP, actin filaments and IP_3Rs associate near the PM.
Figure on preceding page.

a, KRAP was first identified as a cytosolic protein upregulated in cells with constitutively active KRas[33]. KRAP binding to IP_3Rs (probably to its N-terminal, which includes the IP_3-binding core [IBC] and suppressor domain [SD]) requires two critical Phe (F) residues towards the N-terminal of KRAP (red). KRAP binding to actin probably involves residues towards its C-terminal (green)[38]. The KRAP gene (originally termed *SSFA2)* is now called *ITPRID2 (IP$_3$R-interacting domain-containing protein 2)*. Three proteins share sequence similarity with KRAP. TESPA1 (thymocyte-expressed positive-selection-associated protein 1) is expressed predominantly in lymphocytes, where it is implicated in PLC-evoked Ca^{2+} signals[42,43], and perhaps in Ca^{2+} transfer from ER to mitochondria[40]. *ITPRID1* (*IP$_3$R-interacting domain-containing protein 1*) encodes a protein of unknown function, but like TESPA1, it has the sequence required for interaction with IP_3Rs. It is not known whether ITPRID1 binds actin or IP_3R. The KRAP gene also encodes CS-1 (cleavage signal 1)[41], which retains the putative actin-binding region of KRAP, but not its IP_3R-binding sequence. **b**, Proteins from the HeLa interactome[27] that interact with actin, KRAP (*SSFA2*) and the indicated IP_3R subtypes (*ITPR1-3*). ANLN, anilin (F-actin-binding protein); CALML3, calmodulin-like protein 3; CAPZA2, capping actin protein of muscle z-line alpha subunit 2 (binds to barbed end of actin); EVPL, envoplakin (component of desmosomes); FLNA, filamin A (anchors membrane proteins to actin); KCTD5, K^+ channel tetramerization domain-containing protein (may associate with E3 ubiquitin ligases); LIMA1, LIM domain and actin-binding protein 1 (inhibits actin depolymerization); MYH9 and MYH10, myosin heavy chains 9 and 10; MYO1C and MYO19, myosins 1C and 19 (unconventional myosins); SYNPO, synaptopodin (enriched at postsynaptic densities). **c**, WB showing expression of actin and KRAP in wild-type (WT) and EGFP-IP_3R HeLa cells. M_r markers (kDa) are shown. Typical of 3 independent analyses. **d**, Confocal sections across an EGFP-IP_3R1 HeLa cell show distributions of KRAP and EGFP-IP_3Rs. The optical section closest to the coverslip (similar to the TIRF field) is at the top of panel. Scale bar,10 µm (2 µm for enlargements of boxed areas: yellow for near-PM; white for perinuclear areas). Colocalized IP_3R (green) and KRAP (magenta) puncta, which appear white in the overlays, occur only in regions close to the PM. Images typical of 10 cells from 5 dishes. Summary in **Fig. 1f. e**, Cumulative frequency distribution shows centre-to-centre distances of each IP_3R punctum and nearest KRAP punctum for the experimental data from a single cell, and after randomly shuffling KRAP puncta within the cell (100 times) and the associated 95% confidence interval. The analysis was performed for all cells analysed (14,886 IP_3R puncta from 22 cells, **Fig. 1h**). For all cells, the experimental data falls outside the 95% confidence interval (CI), indicating that the observed colocalization (puncta with centre-centre separations < 160 nm) is significant ($P < 0.05$). **f**, Confocal images show the distributions of EGFP-IP_3R1, KRAP and actin filaments throughout the cell (200-nm z-sections). Arrows show examples of colocalizations of all three proteins. Scale bars, 10 µm (5 µm in enlargements of boxes). Images typical of 6 cells from 6 independent dishes. Summary in **Fig. 1k**.

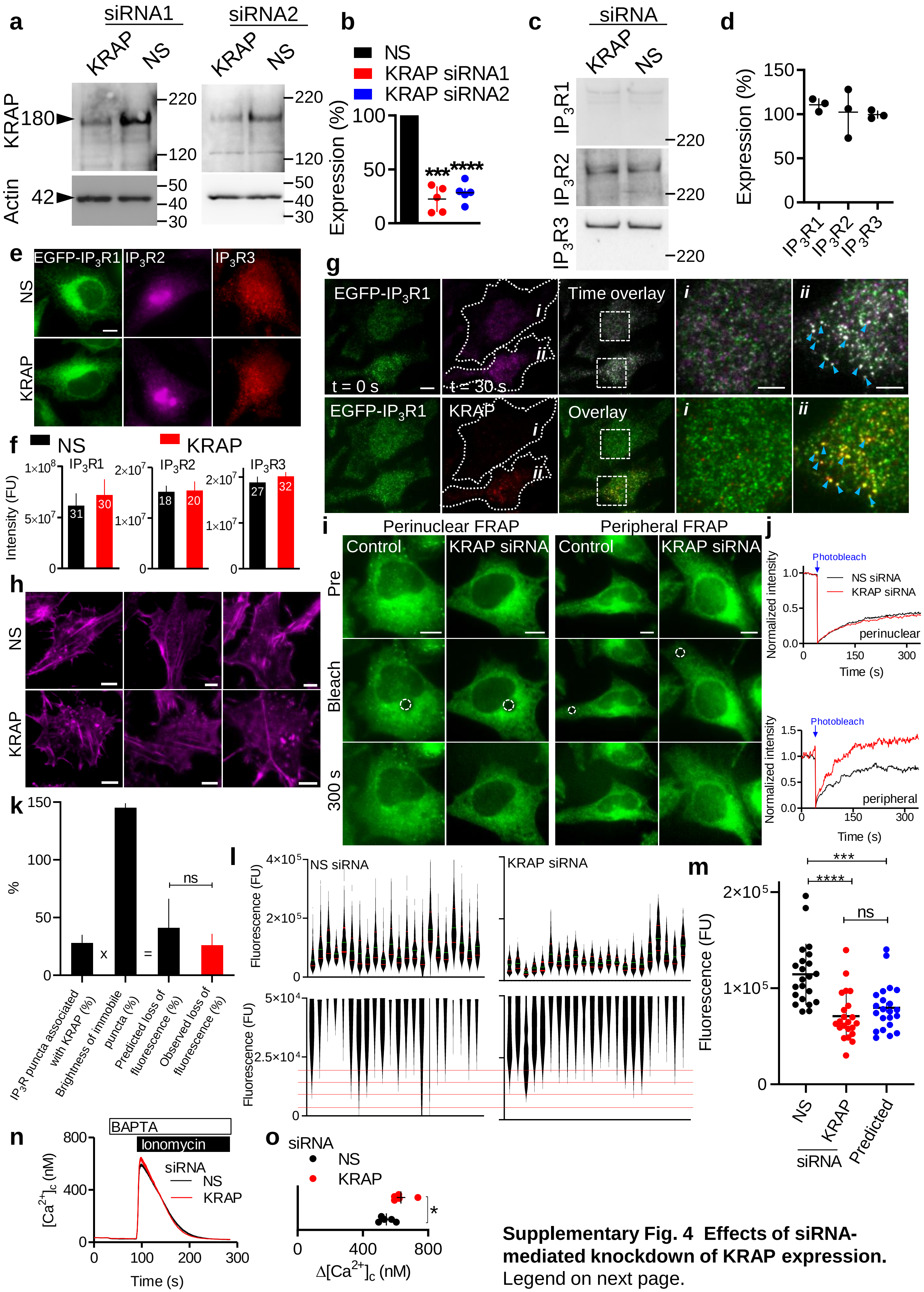

Supplementary Fig. 4 Effects of siRNA-mediated knockdown of KRAP expression. Legend on next page.

Supplementary Fig. 4 Effects of siRNA-mediated knockdown of KRAP expression.
Figure on preceding page.

a, Western blot (WB) showing effects of non-silencing (NS) or two different KRAP siRNAs on KRAP and actin expression in EGFP-IP_3R1 HeLa cells. Positions of calibration standards (kDa) and calculated sizes of bands are shown. **b**, Summary (mean ± s.d., *n* = 5 independent treatments) shows KRAP expression relative to cells treated with NS siRNA $^{***}P < 0.001$, $^{****}P < 0.0001$, paired Student's *t*-test relative to NS siRNA. **c**, WB for each IP_3R subtype after treatment of EGFP-IP_3R1 HeLa cells with the indicated siRNA. Positions of calibration standards (kDa) are shown. **d**, Summary (mean ± s.d., *n* = 3 independent experiments) shows expression (%) relative to matched cells treated with NS siRNA. The 95% CI of the mean for all treatments includes 100%, indicating no significant effect of KRAP siRNA on expression of IP_3R1-3. **e**, Epifluorescence images show effects of KRAP siRNA on EGFP-IP_3R1, and immunostaining for IP_3R2 or IP_3R3. Scale bar, 10 µm. **f**, Summary (mean ± s.d., number of cells (*n*) shown in panels). No significant effect (Student's *t*-test) of KRAP siRNA on IP_3R expression. **g**, TIRF images of cells successfully (*i*) or unsuccessfully (*ii*, a rare example) transfected with KRAP siRNA show immobile IP_3Rs (white) from the time overlay, and examples of colocalization of immobile IP_3Rs with KRAP (arrows). Scale bars, 10 µm (5 µm for enlargements). Images (cell *i*) typical of results from 3 independent dishes. **h**, TIRF images (typical of at least 5 experiments) show actin (Acti-stain 670) in cells treated with NS or KRAP siRNA. Scale bars,10 µm. **i**, EGFP-IP_3R1 HeLa cells were treated with NS or KRAP siRNA, EGFP was focally bleached (diameter 2.3 µm) and FRAP was measured. Epifluorescence images show EGFP fluorescence before, during and 300 s after photobleaching at a perinuclear or peripheral region (dotted circles). Scale bars,10 µm. Images shown are typical of 7-13 cells analysed from 5-6 independent dishes (replicates described fully in summary shown in **Fig. 2j**). **j**, Typical FRAP traces from single cells. Summary in **Fig. 2j**. **k**, Assuming IP_3R puncta are no longer tethered beneath the PM after loss of KRAP, we can predict the loss of fluorescence from puncta in the TIRF field. We begin with the fraction of IP_3R puncta associated with KRAP (**Fig. 1h**, mean ± s.d., *n* = 22 cells) and the fluorescence intensity of immobile relative to mobile puncta (Fig. 2f in[20], mean ± s.d., *n* = 10 cells); and from their product we estimate the predicted loss of TIRF fluorescence (third column, for which the variance is estimated according to[76] (**Supplementary Table 1**). The results (mean ± s.e.m.) show no significant difference (Student's *t*-test) between the observed (fourth column, mean ± s.e.m., *n* = 22 cells) and predicted loss of fluorescence of IP_3R puncta in the TIRF field (**Fig. 2k**). **l**, Violin plots show fluorescence intensities of all IP_3R puncta in the TIRF field for 22 cells (with 407-1299 puncta analysed in each) treated with NS or KRAP siRNA. Red bars show 25th and 75th percentiles and green bars show medians. Lower panels show the lowest fluorescent intensities and demonstrate that there is no evident appearance of unusually small puncta after loss of KRAP. Summary in **Fig. 2l**. **m**, 28% of IP_3R puncta associate with KRAP (**Fig. 1h**), if we assume these are the brightest puncta, we can estimate the consequences of losing KRAP-associated IP_3Rs on the mean fluorescence intensity of IP_3R puncta. Results (22 cells) show mean fluorescence intensities of IP_3R puncta for cells treated with NS or KRAP siRNA (data in **Fig**. **2l**) and the predicted values after removal of the brightest 28% of puncta from cells treated with NS siRNA. Mean ± s.e.m., $^{****}P < 0.0001$, $^{***}P < 0.001$, ns $P > 0.05$, one-way ANOVA with Bonferroni's test. These results (k-m) indicate that KRAP is unlikely to assemble IP_3R puncta, but instead anchors pre-assembled puncta on actin beneath the PM (**Fig. 2n**). **n**, Effects of siRNA on $[Ca^{2+}]_c$ after addition of BAPTA (2.5 mM) to chelate extracellular Ca^{2+}, and then ionomycin (5 µM) to release Ca^{2+} from intracellular stores. Traces show average results from 6 wells in a single experiment. **o**, Summary (mean ± s.e.m., *n* = 5, each with 6 replicates) shows peak increase in $[Ca^{2+}]_c$ ($\Delta[Ca^{2+}]_c$) evoked by ionomycin**.** $^{*}P < 0.05$, Student's *t*-test.

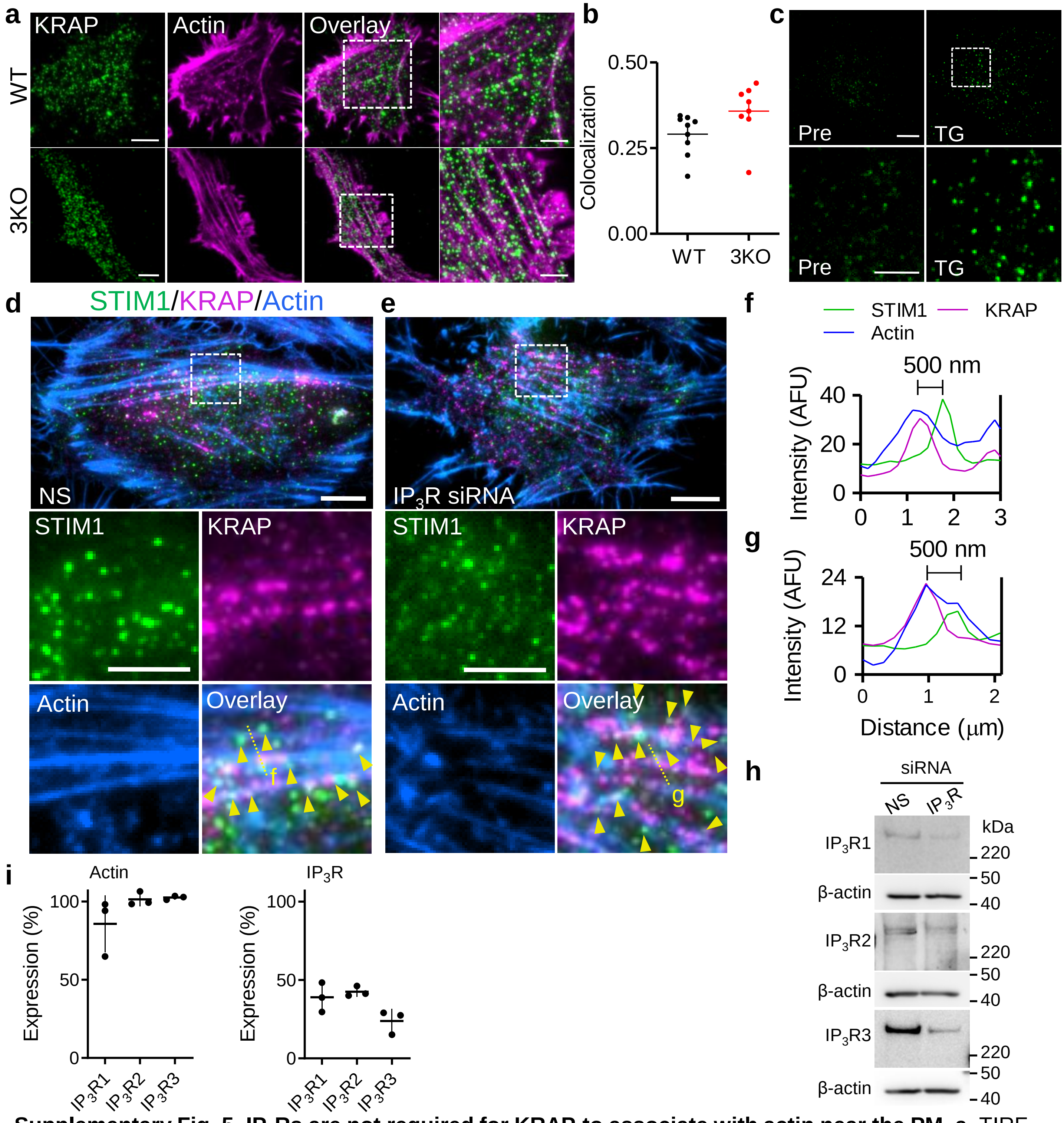

Supplementary Fig. 5 IP$_3$Rs are not required for KRAP to associate with actin near the PM. a, TIRF images of cells stained for KRAP and actin (Acti-stain 670) show that KRAP localizes to actin filaments similarly in WT HEK cells and in cells devoid of IP$_3$Rs (3KO). Scale bars,10 µm (5 µm in enlargements). **b**, Summary shows Mander's split coefficient for KRAP relative to actin filaments; mean ± s.d., *n* = 9 (WT) or 8 (3KO) cells, *P* > 0.05, Student's *t*-test. **c**, TIRF images of a STIM1-EGFP HeLa cell before and after treatment in Ca^{2+}-free HBS with thapsigargin (TG, 1 µM, 5 min) to deplete the ER of Ca^{2+}. Boxed area enlarged in the bottom panels. Scale bar, 10 µm (5 µm in enlargements). Typical of 3 independent analyses. **d**,**e**, TIRF images of STIM1-EGFP HeLa cells treated with NS siRNA (d) or siRNA to all three IP$_3$R subtypes (e), and then thapsigargin (1 µM, 15 min) to empty Ca^{2+} stores, before fixation and labelling of actin (Acti-stain 670) and KRAP. Scale bar,10 µm (2 µm in enlargements). Results typical of 9 cells from 5 experiments (d) or 10 cells from 6 experiments (e). Arrows highlight juxtapositions of actin-associated KRAP and STIM1 puncta. **f**,**g**, Fluorescence intensity profiles of actin, STIM1 and KRAP along lines shown in d and e show that the STIM1 and KRAP are juxtaposed rather than colocalized. AFU, arbitrary fluorescence units. Summary in **Fig. 2o**. **h**, WB showing expression of actin and IP$_3$R subtypes in STIM1-EGFP HeLa cells after treatment with siRNA directed to each of the three IP$_3$R subtypes and NS siRNA. **i**, Summary (mean ± s.d., *n* = 3 independent treatments) shows expression (%) of each IP$_3$R subtype or actin, relative to cells treated with NS siRNA. The 95% CI of the mean for all actin measurements included 100% (indicating no significant effect of IP$_3$R siRNAs on actin expression). All 95% CI for measurements of IP$_3$R expression excluded 100% (indicating a significant effect, *P* < 0.05, of IP$_3$R siRNAs on IP$_3$R expression).

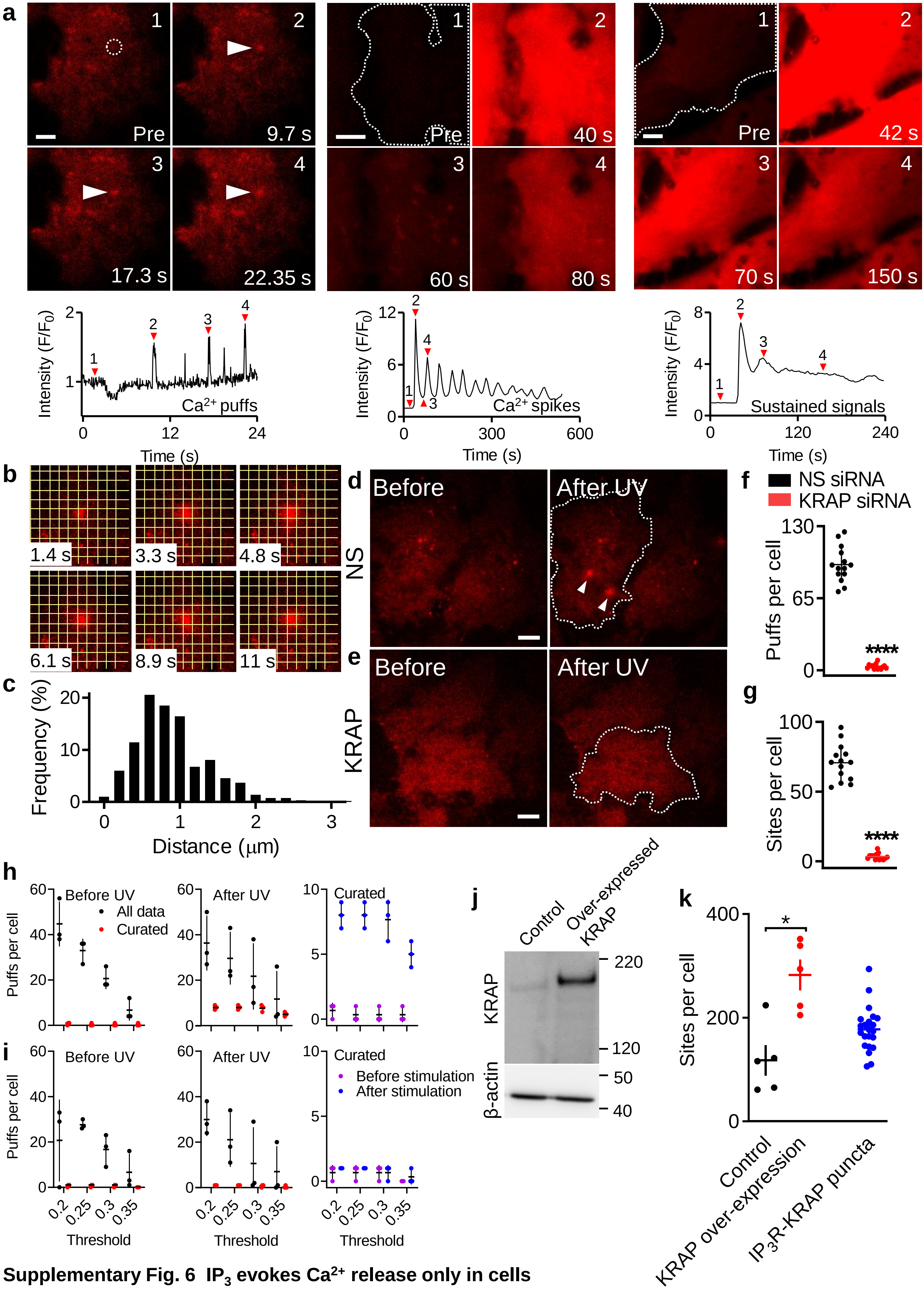

Supplementary Fig. 6 IP$_3$ evokes Ca^{2+} release only in cells expressing KRAP.
Legend on next page.

Supplementary Fig. 6 IP_3 evokes Ca^{2+} release only in cells expressing KRAP.
Figure on preceding page.

a, TIRFM was used to record Ca^{2+} signals (Cal-590) from EGFP-IP_3R1 HeLa cells (typically 5-7 cells per field), loaded with EGTA (by incubation with EGTA-AM, to constrain propagation of Ca^{2+} signals), and then stimulated with histamine. For each cell, the first discernible response to histamine was categorized as a Ca^{2+} puff, Ca^{2+} spike (at least two transient global increases in fluorescence (F) with F returning to a level no greater than 2x basal F between transients) or a sustained global increase in F. Time courses of Cal-590 fluorescence from the indicated regions illustrate the three categories of response. Accompanying TIRF images are taken from the times indicated (1-4). Scale bars, 5 µm. The acquisition rates required to record Ca^{2+} puffs (20 Hz) and Ca^{2+} spikes (0.2 Hz) differ; we therefore overestimate the number of cells in which Ca^{2+} spikes were the first response since preceding Ca^{2+} puffs may pass undetected (hence, the percentage responses do not sum to 100%). This problem does not affect the relative abundance of Ca^{2+} spikes and sustained Ca^{2+} signals, with the latter requiring higher concentrations of histamine. Summary in **Fig. 3a**. **b**, Examples of six Ca^{2+} puffs evoked by photolysis (150 ms) of ci-IP_3 occurring at the same site. The grid shows 2 µm squares. Times after UV flash are shown. **c**, Distribution of distances between centroids of the first Ca^{2+} puff at a site and each successive Ca^{2+} puff at the same site (1024 puffs from 341 sites in 19 cells). Summary in **Fig. 3f**. **d**,**e**, TIRF images of Ca^{2+} signals before and after photolysis (150 ms) of ci-IP_3 in cells treated with NS (d) or KRAP siRNA (e). Scale bars, 10 µm. **f**,**g**, Summary (mean ± s.d., 14 cells) show numbers of Ca^{2+} puffs (f) and sites (g) per cell detected in the 20 s after photolysis of ci-IP_3. $^{****}P < 0.0001$, Student's t-test. **h**,**i,** Effects of adjusting threshold settings in the FLIKA algorithm used for automated detection of Ca^{2+} puffs[72] on numbers of Ca^{2+} puffs detected during 2.5-s recordings before and beginning 2.5 s after UV-photolysis of ci-IP_3. Results show all puffs identified by FLIKA and the number detected after manual inspection of all identified puffs; this curation was applied to all analyses reported in the present study. Results (mean ± s.d.) are from 3 cells treated with NS (h) or KRAP siRNA (i). The results confirm that the threshold setting used for our analyses (0.3) is optimal for detecting all Ca^{2+} puffs that pass manual inspection criteria with the fewest false-positives. More importantly, the results demonstrate that in cells lacking KRAP, the numbers of Ca^{2+} puffs identified by FLIKA at all thresholds are indistinguishable before and after photolysis of ci-IP_3. Furthermore, there is no difference in the very small numbers of Ca^{2+} puffs detected after curation before and after photolysis of ci-IP_3 in KRAP-depleted cells. We conclude that loss of KRAP does not cause IP_3-evoked Ca^{2+} puffs to become smaller. **j**, Western blot showing effects of over-expressing KRAP in EGFP-IP_3R1 HeLa cells (see **Fig. 4a-g**). Calibration markers (kDa) are shown. In 3 independent analyses, KRAP expression was 9.5 ± 1.1 (mean ± s.d.)-fold greater in cells over-expressing KRAP. **k**, Since Ca^{2+}-release sites are identified only when they evoke a Ca^{2+} puff, we are more likely to miss sites when puffs are infrequent. We have reported methods to estimate the number of 'missed' sites and so estimate the total number of sites (N) from the smaller number observed in recordings of fixed duration[16]. The method used here examines the number of puffs occurring at each identified site during either the 20 s after photolysis of ci-IP_3 (control) or until the Ca^{2+} signals propagate globally (5-7 s, KRAP over-expression). It then uses the Poisson Distribution to estimate N from the mean number of Ca^{2+} puffs/site (µ), the number of sites at which only a single puff occurred (Obs_1), and the number of sites at which two puffs occurred (Obs_2): $\mu = 2Obs_2 / Obs_1$, and $N = Obs_1 / \mu.e^{-\mu}$. Results show the data used to estimate N for control cells and cells over-expressing KRAP (from **Fig. 4d**). Summary results show mean ± s.e.m., and values from individual cells (n = 5 cells for control and KRAP-over-expression, n = 22 cells for IP_3R-KRAP puncta). $^{*}P < 0.05$, Student's unpaired t-test. The analyses confirm that Ca^{2+} puffs initiate at more sites in cells over-expressing KRAP. Similar results were obtained when the analysis was restricted to Ca^{2+} puffs detected in the 5-s interval after photolysis of ci-IP_3: N = 85 ± 33 for control, and 279 ± 53 for cells over-expressing KRAP. It was impracticable to apply similar analyses to cells treated with KRAP siRNA because Ca^{2+} puffs were so infrequent that they were only very rarely detected at the same site (**Fig. 3h**). For comparison, numbers of colocalized IP_3R-KRAP puncta per cell are also shown for control cells (n = 22 cells).

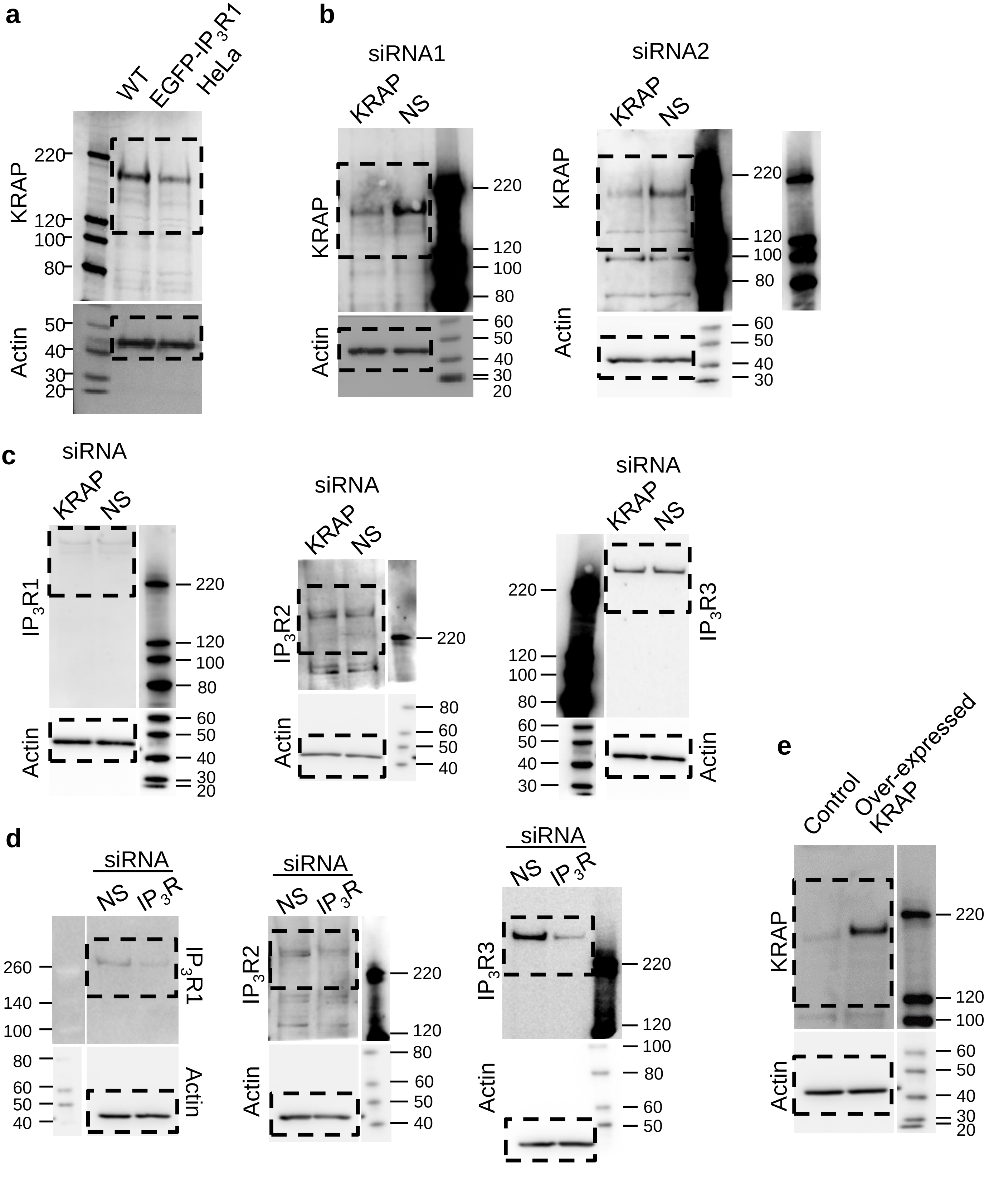

Supplementary Fig. 7 Uncropped images of Western blots. a, WB from **Supplementary Fig. 3c**. **b**, WB from **Supplementary Fig. 4a. c**, WB from **Supplementary Fig. 4c. d**, WB from **Supplementary Fig. 5h. e**, WB from **Supplementary Fig. 6j**. M_r markers (kDa) are shown for each uncropped WB. The primary antibodies used were: KRAP (Proteintech, cat # 14157-1-AP; RRID, AB_2195472) (a, b, e); IP_3R1 (Merck Millipore, cat # AB5882; RRID, AB_92112 and AB_92113) (c, d); IP_3R2 (custom made by Pocono Rabbit Farm) (c, d); and IP_3R3 (BD Transduction Laboratories, cat # 610313; RRID, AB_397705) (c, d).

Supplementary Table 1 Summary of statistical analyses.

$^{*}P < 0.05$; $^{**}P < 0.01$; $^{***}P < 0.001$; $^{****}P < 0.0001$

<table>
<tr><th>Figure</th><th>Test</th><th>P values and summary</th></tr>
<tr><td colspan="3">Main figures</td></tr>
<tr><td>Fig. 1a, puffs</td><td>2-tailed unpaired Student’s t-test</td><td>P = 0.9774 (ns)</td></tr>
<tr><td>Fig. 1a, sites</td><td>2-tailed unpaired Student’s t-test</td><td>P = 0.9999 (ns)</td></tr>
<tr><td>Fig. 1b</td><td>2-tailed unpaired Student’s t-test</td><td>P = 0.9970 (ns)</td></tr>
<tr><td>Fig. 1d</td><td>2-tailed unpaired Student’s t-test</td><td>P < 0.0001 (****)</td></tr>
<tr><td>Fig. 1f</td><td>Ordinary one-way ANOVA then Bonferroni’s multiple comparisons test</td><td>P = 0.0247 (ANOVA). Bonferroni’s multiple comparisons test for all comparisons:
<table>
<tr><th></th><th>Adjusted P value</th><th>P</th></tr>
<tr><td>Near-PM (black) vs middle/periphery (blue)</td><td>0.1513</td><td>ns</td></tr>
<tr><td>Near-PM (black) vs middle/centre (red)</td><td><0.0001</td><td>P < 0.0001 (****)</td></tr>
<tr><td>Middle/periphery (blue) vs middle/centre (red)</td><td>0.0040</td><td>P < 0.01 (**)</td></tr>
</table></td></tr>
<tr><td>Fig. 1h</td><td>Costes randomization test, then 2-tailed unpaired Student’s t-test</td><td>Costes randomization (see Supplementary Fig. 3e) was used to provide 95% CI for the randomized distribution for each cell. Observed separations fall outside this 95% CI for all cells, indicating that the observed separations of IP_3R puncta and KRAP puncta are significantly different from the randomized distribution (P < 0.05).
Puncta separated by ≤ 160 nm are considered to be co-localized. For each of 22 cells, we established the fraction of IP_3R puncta that satisfied the colocalization criterion and compared observed values (black) with those determined after randomization of the KRAP distribution (magenta). Summary results are reported in the legend to Fig. 1h. Student’s t-test, P < 0.0001 (****).</td></tr>
<tr><td>Fig. 1k</td><td>2-tailed unpaired Student’s t-test</td><td>P < 0.0001 (****)</td></tr>
<tr><td>Fig. 2i</td><td>2-tailed unpaired Student’s t-test</td><td>P < 0.0001 (****)</td></tr>
<tr><td>Fig. 2j, perinuclear</td><td>2-tailed unpaired Student’s t-test</td><td>P = 0.7408 (ns)</td></tr>
<tr><td>Fig. 2j, periphery</td><td>2-tailed unpaired Student’s t-test</td><td>P = 0.0040 (**)</td></tr>
<tr><td>Fig. 2k</td><td>2-tailed unpaired Student’s t-test</td><td>P = 0.0371 (*)</td></tr>
<tr><td>Fig. 2l</td><td>2-tailed unpaired Student’s t-test</td><td>P < 0.0001 (****)</td></tr>
<tr><td>Fig. 2m</td><td>2-tailed unpaired Student’s t-test</td><td>P = 0.0034 (**)</td></tr>
<tr><td>Fig. 2o</td><td>Costes randomization</td><td>Costes randomization (with KRAP puncta randomized) provides 95% CI for the randomized data. For each cell, the observed values for the cumulative frequency distributions lie outside the 95% CI for the distributions after randomization (P < 0.05).</td></tr>
<tr><td>Fig. 3h</td><td>Ordinary one-way ANOVA then Bonferroni’s multiple comparisons test</td><td>P < 0.0001 (ANOVA). Bonferroni’s multiple comparisons test for all comparisons:
<table>
<tr><th></th><th>Adjusted P value</th><th>P</th></tr>
<tr><td>NS siRNA vs KRAP siRNA</td><td><0.0001</td><td>P < 0.0001 (****)</td></tr>
<tr><td>NS siRNA vs rescue</td><td>>0.9999</td><td>ns</td></tr>
<tr><td>KRAP siRNA vs rescue</td><td><0.0001</td><td>P < 0.0001 (****)</td></tr>
</table></td></tr>
</table>

<table>
<tr><td>Fig. 3i</td><td>Ordinary one-way ANOVA then Bonferroni’s multiple comparisons test</td><td>$P < 0.0001$ (ANOVA). Bonferroni’s multiple comparisons test for all comparisons:
<table>
<tr><td></td><td>Adjusted P value</td><td>P</td></tr>
<tr><td>NS siRNA vs KRAP siRNA</td><td><0.0001</td><td>$P < 0.0001$ (****)</td></tr>
<tr><td>NS siRNA vs rescue</td><td>>0.9999</td><td>ns</td></tr>
<tr><td>KRAP siRNA vs rescue</td><td><0.0001</td><td>$P < 0.0001$ (****)</td></tr>
</table></td></tr>
<tr><td>Fig. 3k</td><td>Ordinary one-way ANOVA then Bonferroni’s multiple comparisons test</td><td>$P < 0.0002$ (ANOVA). Bonferroni’s multiple comparisons test for all comparisons:
<table>
<tr><td></td><td>Adjusted P value</td><td>P</td></tr>
<tr><td>NS siRNA vs KRAP siRNA</td><td>0.0115</td><td>$P < 0.05$(*)</td></tr>
<tr><td>NS siRNA vs rescue</td><td>0.1495</td><td>ns</td></tr>
<tr><td>KRAP siRNA vs rescue</td><td>0.0001</td><td>$P < 0.001$ (***)</td></tr>
</table></td></tr>
<tr><td>Fig. 3m</td><td>2-tailed unpaired Student’s t-test</td><td>$P < 0.0001$ (****)</td></tr>
<tr><td>Fig. 3n</td><td>2-tailed unpaired Student’s t-test</td><td>$P < 0.0001$ (****)</td></tr>
<tr><td>Fig. 4b</td><td>2-tailed unpaired Student’s t-test</td><td>$P = 0.0031$ (**)</td></tr>
<tr><td>Fig. 4d, puffs</td><td>2-tailed unpaired Student’s t-test</td><td>$P = 0.0004$ (***)</td></tr>
<tr><td>Fig. 4d, sites</td><td>2-tailed unpaired Student’s t-test</td><td>$P = 0.0008$ (***)</td></tr>
<tr><td>Fig. 4f</td><td>2-tailed unpaired Student’s t-test</td><td>$P = 0.4134$ (ns)</td></tr>
<tr><td>Fig. 4g, right panel</td><td>2-tailed unpaired Student’s t-test</td><td>$P = 0.0470$ (*)</td></tr>
<tr><td colspan="3">Supplementary Figs</td></tr>
<tr><td>Fig. 2c, actin</td><td>2-tailed unpaired Student’s t-test, observed vs randomized</td><td>$P < 0.0001$ (****)</td></tr>
<tr><td>Fig. 2c, vimentin</td><td>2-tailed unpaired Student’s t-test, observed vs randomized</td><td>$P = 0.2553$ (ns)</td></tr>
<tr><td>Fig. 3e</td><td>Costes randomization</td><td>Costes randomization was used to provide the 95% CI for the randomized cumulative frequency distribution. For all cells ($n = 22$) the observed separations fall outside this 95% CI, indicating that the observed separations of IP_3R puncta and KRAP puncta are significantly different from the randomized distribution ($P < 0.05$).</td></tr>
<tr><td>Fig. 4b,
NS vs KRAP siRNA1</td><td>2-tailed paired Student’s t-test</td><td>$P = 0.001$ (***)</td></tr>
<tr><td>Fig. 4b,
NS vs KRAP siRNA2</td><td>2-tailed paired Student’s t-test</td><td>$P = 0.0001$ (****)</td></tr>
</table>

<table>
<tr><td>Fig. 4d</td><td>Comparison of 95% CI with 100%</td><td>Mean and (95% CI of mean):
IP_3R1, 110.6 (92.34 to 128.9) (ns)
IP_3R2, 102.3 (33.53 to 171.0) (ns)
IP_3R3, 99.41 (87.97 to 110.9) (ns)</td></tr>
<tr><td>Fig. 4f, IP_3R1</td><td>2-tailed unpaired Student’s t-test</td><td>$P = 0.5949$ (ns)</td></tr>
<tr><td>Fig. 4f, IP_3R2</td><td>2-tailed unpaired Student’s t-test</td><td>$P = 0.9029$ (ns)</td></tr>
<tr><td>Fig. 4f, IP_3R3</td><td>2-tailed unpaired Student’s t-test</td><td>$P = 0.4161$ (ns)</td></tr>
<tr><td>Fig. 4k</td><td>2-tailed unpaired Student’s t-test</td><td>Variance (var_{xy}) of the predicted loss of fluorescence (third column) computed from var_x and μ_x (first column) and var_y and μ_y (second column) according to[76]: $var_{xy} = \mu_x^2 var_y + \mu_y^2 var_x$.
2-tailed unpaired Student’s t-test for comparison of predicted (third column) and observed (forth column) fluorescence, $P = 0.543$ (ns).</td></tr>
<tr><td>Fig. 4m</td><td>Ordinary one-way ANOVA then Bonferroni’s multiple comparisons test</td><td>$P < 0.0001$ (ANOVA). Bonferroni’s multiple comparisons test for all comparisons:
<table>
<tr><td></td><td>Adjusted P value</td><td>P</td></tr>
<tr><td>NS siRNA vs KRAP siRNA</td><td>0.0001</td><td>$P < 0.0001$(****)</td></tr>
<tr><td>NS siRNA vs predicted</td><td>0.0003</td><td>$P < 0.001$(***)</td></tr>
<tr><td>KRAP siRNA vs predicted</td><td>0.8540</td><td>ns</td></tr>
</table></td></tr>
<tr><td>Fig. 4o</td><td>2-tailed unpaired Student’s t-test</td><td>$P = 0.0280$ (*)</td></tr>
<tr><td>Fig. 5b</td><td>2-tailed unpaired Student’s t-test</td><td>$P = 0.0680$ (ns)</td></tr>
<tr><td>Fig. 5i, actin</td><td>Comparison of 95% CI with 100%</td><td>Mean and (95% CI of mean):
IP_3R1, 85.77 (40.50 to 131.0 (ns)
IP_3R2, 101.4 (90.47 to 112.4) (ns)
IP_3R3, 102.5 (99.5 to 105.5) (ns)</td></tr>
<tr><td>Fig. 5i, IP_3R</td><td>Comparison of 95% CI with 100%</td><td>Mean and (95% CI of mean):
IP_3R1, 38.96 (15.75 to 62.17 (*)
IP_3R2, 42.53 (34.48 to 50.57) (*)
IP_3R3, 23.88 (4.957 to 42.81) (*)</td></tr>
<tr><td>Fig. 6f</td><td>2-tailed unpaired Student’s t-test</td><td>$P = 0.0001$ (****)</td></tr>
<tr><td>Fig. 6g</td><td>2-tailed unpaired Student’s t-test</td><td>$P = 0.0001$ (****)</td></tr>
<tr><td>Fig. 6k</td><td>2-tailed unpaired Student’s t-test</td><td>Comparison of control (black) with KRAP over-expression (red) only: $P = 0.0299$ (*)</td></tr>
</table>
