# Supplementary material for: KRAP tethers IP3 receptors to actin and licenses them to evoke cytosolic Ca2+ signals
Source: Nat Commun. 2021 Jul 23;12:4514. doi: 10.1038/s41467-021-24739-9 (PMC8302619; doi:10.1038/s41467-021-24739-9)
Supplement: Supplementary file 13 — Description of additional supplementary files [file 41467_2021_24739_MOESM13_ESM.docx]

Description of additional supplementary files

Title: Supplementary Movie 1

Description; A subset of IP3Rs colocalize with actin filaments. EGFP-IP3R1 HeLa cells were fixed and stained with Actistain 670 to reveal actin filaments. 3D projections of confocal images (200-nm steps in z-plane) show actin filaments alone (magenta) and overlaid with IP3Rs (green, right panel). A subset of IP3R puncta (namely those that are immobile and immediately beneath the PM) colocalize with actin filaments.

Title: Supplementary Movie 2

Description; Immobile IP3R puncta retreat with actin filaments after treatment with cytochalasin D. EGFP-IP3R1 HeLa cells expressing LifeAct-mCherry (to identify actin) were treated with cytochalasin D (10 µM, added at 0.5 min) to depolymerize actin filaments. Time-lapse TIRFM images show IP3Rs (green, left panel), actin filaments (red, middle) and their overlay (right). As the actin filaments depolymerize, immobile IP3R puncta retreat with the filaments and become concentrated around residual filaments (see, for example, filaments at left retracting upwards, and in the middle filaments retracting downwards). Images acquired at 6 frames per min and displayed at 15 frames per s (fps). Time shown as hr:min:s.ms.

Title: Supplementary Movie 3

Description: Immobile IP3R puncta retreat with actin filaments after treatment with latrunculin A. EGFP-IP3R1 HeLa cells expressing LifeAct-mCherry were treated with latrunculin A (5 µM, added at 0.5 min). TIRFM images show IP3Rs (green), actin filaments (red) and their overlay. As the actin filaments depolymerize, immobile IP3R puncta retreat with the filaments and become concentrated around residual actin filaments (see, for example, the central filaments (from ~9 min onwards) as they retract upwards). Images acquired at 6 frames per min and displayed at 15 fps. Time shown as hr:min:s.ms.

Title: Supplementary Movie 4

Description: Immobile IP3R puncta do not clearly associate with vimentin. EGFP-IP3R1 HeLa cells expressing mCherry-vimentin were treated with latrunculin A to depolymerize actin (5 µM, added at 0.5 min). Overlaid time-lapse TIRF images of IP3Rs (green) and vimentin intermediate filaments (red). There are many immobile IP3R puncta in regions of the cell apparently devoid of vimentin (see, for example, much of the lower portion of the cell). As actin filaments retract after addition of latrunculin A, several immobile IP3R puncta are dragged (presumably through their association with actin, Supplementary Movies 2, 3) into regions without vimentin (see, for example, the string of puncta immediately left of the densest vimentin, which move (at ~11 min) into a region without vimentin). Images acquired at 6 frames per min and displayed at 10 fps. Time shown as hr:min:s.ms.

Title: Supplementary Movie 5

Description: A subset of near-PM IP3R puncta colocalize with KRAP. EGFP-IP3R1 HeLa cells were fixed and immunostained with an antibody to KRAP. 3D projections of confocal images (200-nm steps in z-plane) of IP3Rs (green), KRAP (red,) and their overlay show that a subset of IP3R puncta colocalize with a subpopulation of KRAP puncta near the PM. The linear arrays of colocalized proteins (yellow) is consistent with their association with actin (Supplementary Movies 1-3).

Title: Supplementary Movie 6

Description: IP3Rs and KRAP colocalize at regions of actin filaments. EGFP-IP3R1 HeLa cells were fixed and immunostained for KRAP and stained for actin filaments (Acti-stain 670). 3D projections of confocal images (200-nm steps in z-plane) show overlays of IP3Rs with actin (green and magenta) or KRAP (green and red). A subset of IP3R puncta colocalize with KRAP, but only in association with actin.

Title: Supplementary Movie 7

Description: IP3R puncta are no longer immobile after KRAP knockdown. Time-lapse TIRFM images of EGFP-IP3R1 HeLa cells treated with siRNA against KRAP (right) or a non-silencing siRNA (control, left). Images acquired at 2 fps and displayed at 10 fps. Time shown as hr:min:s.ms. Loss of KRAP causes almost complete loss of immobility in near-PM IP3R puncta.

Title: Supplementary Movie 8

Description: Loss of KRAP almost abolishes IP3- evoked Ca2+ puffs. TIRFM images of EGFP-IP3R1 HeLa cells treated with siRNA against KRAP or a non-silencing siRNA (control) were loaded with a Ca2+ indicator (Cal-590), EGTA and ci-IP3. Photolysis of the ci-IP3 (150-ms UV flash, at 2.5 s) evokes Ca2+ puffs in control cells (left), but not in cells lacking KRAP (right). Images acquired and displayed at 20 fps. Time shown as hr:min:s.ms.

Title: Supplementary Movie 9

Description: Cal-590 fluorescence recorded from cells lacking KRAP before and after photolysis of ci-IP3. TIRFM images of EGFP-IP3R1 HeLa cells treated with siRNA against KRAP were loaded with a Ca2+ indicator (Cal-590), EGTA and ci-IP3. Movies show fluorescence recorded from 3 cells during 2.5-s intervals immediately before and beginning 2.5 s after photolysis of ci-IP3 (150- ms UV flash). Images acquired at 20 fps and displayed at 10 fps. Time shown as hr:min:s.ms.

Title: Supplementary Movie 10

Description: Over-expressed KRAP increases the frequency of IP3-evoked Ca2+ puffs. EGFP-IP3R1 HeLa cells mocktransfected (control, left) or transfected with plasmid encoding KRAP (KRAP over-expression, right) were loaded with a Ca2+ indicator (Cal- 590), EGTA and ci-IP3. Photolysis of ci-IP3 (150-ms UV flash, at 2.5 s) evokes Ca2+ puffs that are more abundant and then rapidly propagate into global Ca2+ signals in cells over-expressing KRAP. Images acquired and displayed at 20 fps. Time shown as hr:min:s.ms.
