# Supplementary material for: KRAP tethers IP3 receptors to actin and licenses them to evoke cytosolic Ca2+ signals
Source: Nat Commun. 2021 Jul 23;12:4514. doi: 10.1038/s41467-021-24739-9 (PMC8302619; doi:10.1038/s41467-021-24739-9)
Supplement: Supplementary file 14 — Reporting Summary [file 41467_2021_24739_MOESM14_ESM.pdf]

nature research

Corresponding author(s): Taylor, CW & Thillaiappan, NB

Last updated by author(s): Jun 4, 2021

Reporting Summary

Nature Research wishes to improve the reproducibility of the work that we publish. This form provides structure for consistency and transparency in reporting. For further information on Nature Research policies, see our Editorial Policies and the Editorial Policy Checklist.

Statistics

For all statistical analyses, confirm that the following items are present in the figure legend, table legend, main text, or Methods section.

n/a	Confirmed	
☐	☒	The exact sample size (*n*) for each experimental group/condition, given as a discrete number and unit of measurement
☐	☒	A statement on whether measurements were taken from distinct samples or whether the same sample was measured repeatedly
☐	☒	The statistical test(s) used AND whether they are one- or two-sided<br>*Only common tests should be described solely by name; describe more complex techniques in the Methods section.*
☒	☐	A description of all covariates tested
☐	☒	A description of any assumptions or corrections, such as tests of normality and adjustment for multiple comparisons
☐	☒	A full description of the statistical parameters including central tendency (e.g. means) or other basic estimates (e.g. regression coefficient) AND variation (e.g. standard deviation) or associated estimates of uncertainty (e.g. confidence intervals)
☐	☒	For null hypothesis testing, the test statistic (e.g. *F*, *t*, *r*) with confidence intervals, effect sizes, degrees of freedom and *P* value noted<br>*Give P values as exact values whenever suitable.*
☒	☐	For Bayesian analysis, information on the choice of priors and Markov chain Monte Carlo settings
☒	☐	For hierarchical and complex designs, identification of the appropriate level for tests and full reporting of outcomes
☒	☐	Estimates of effect sizes (e.g. Cohen's *d*, Pearson's *r*), indicating how they were calculated

Our web collection on statistics for biologists contains articles on many of the points above.

Software and code

Policy information about availability of computer code

Data collection	All software used for data collection is cited in the manuscript with citations [Metamorph v7.10.1.161, SoftMax Pro v5.4]. No custom unpublished software was used.
Data analysis	All software used for data analysis is reported in the manuscript with citations [PRISM v6, Metamorph v7.10.1.161, Fiji v1.51n (TrackMate v3.8, FRAP profiler (version not provided), TrajClassifier v0.83, Microvolution deconvolution algorithm v2015.05), DiAna v1.1, SoftMax Pro v5.4, FLIKA v1]. No custom unpublished software was used.

For manuscripts utilizing custom algorithms or software that are central to the research but not yet described in published literature, software must be made available to editors and reviewers. We strongly encourage code deposition in a community repository (e.g. GitHub). See the Nature Research guidelines for submitting code & software for further information.

Data

Policy information about availability of data

All manuscripts must include a data availability statement. This statement should provide the following information, where applicable:

- Accession codes, unique identifiers, or web links for publicly available datasets
- A list of figures that have associated raw data
- A description of any restrictions on data availability

All data required to support the conclusions of this paper are provided in Figs 1-5, Supplementary Figs 1-7, Supplementary Table 1, Supplementary Movies 1-10 and the Data Resource File. All software and algorithms used are freely available from sources listed in Methods. Materials and primary data are available from the corresponding authors upon reasonable request.

Field-specific reporting

Please select the one below that is the best fit for your research. If you are not sure, read the appropriate sections before making your selection.

☒ Life sciences ☐ Behavioural & social sciences ☐ Ecological, evolutionary & environmental sciences

For a reference copy of the document with all sections, see nature.com/documents/nr-reporting-summary-flat.pdf

Life sciences study design

All studies must disclose on these points even when the disclosure is negative.

Sample size	No statistical methods were used to predetermine sample sizes. The smallest number of independent replicates reported is for WB (n=3), which have historically provided sufficient consistency to support the conclusions presented. It was impracticable, with entirely novel data, to complete formal power analyses before deciding sample sizes. All sample sizes were dictated by feasibility; they were not modified after statistical analysis.
Data exclusions	No data were excluded.
Replication	All experiments were replicated,with the biological replicates results (figure legends report sample sizes for technical and biological replicates). All results report the analysis of at least 3 independent replicates.
Randomization	There was no need for systematic randomization of samples because most treatments were of parallel preparations of cells in which the only variable was the treatment. Some statistical analyses are supported by illustrative traces,which were randomly selected from an Excel Spreadsheet that include all cells captured within the field [eg Fig. 3l). Observer bias in analysis of images is avoided by pre-determined quantitative analysis.
Blinding	Blinding was not required for this study because there were no data exclusions and all conclusions reset on objective quantitative analysis of data extracted from images.

Reporting for specific materials, systems and methods

We require information from authors about some types of materials, experimental systems and methods used in many studies. Here, indicate whether each material, system or method listed is relevant to your study. If you are not sure if a list item applies to your research, read the appropriate section before selecting a response.

Materials & experimental systems

n/a	Involved in the study	
☐	☒	Antibodies
☐	☒	Eukaryotic cell lines
☒	☐	Palaeontology and archaeology
☒	☐	Animals and other organisms
☒	☐	Human research participants
☒	☐	Clinical data
☒	☐	Dual use research of concern

Methods

n/a	Involved in the study	
☒	☐	ChIP-seq
☒	☐	Flow cytometry
☒	☐	MRI-based neuroimaging

Antibodies

Antibodies used	Anti-β-actin (mouse monoclonal; Cell Signaling Technology, Leiden,Netherlands,#8H1OD10, undefined Clone #).<br>Anti-KRAP (rabbit polyclonal; ProteinTech, Manchester, UK, #14157-1-AP), the same anti-KRAP primary antibody was also custom-conjugated to YF-594 (ProteinTech).<br>GFP Tag-AlexaFluor-647 (STORM, ThermoFisher, #31852).<br>Anti-IP3R1 (rabbit, C-terminal peptide 2732–2750 of rat IP3R1;Merck Millipore, #AB5882).<br>Anti-IP3R2 (rabbit, custom-made to a C-terminal peptide GFLGSNTPHENHHMPPH; Pocono Rabbit Farm and Laboratory) Ref 41.<br>Anti-IP3R3 (mouse monoclonal; BD Transduction Laboratories, Wokingham, UK, #610313, Clone 2).<br>Anti-STIM1 (rabbit monoclonal; Cell Signaling Technology, #5668, undefined Clone #)<br>Anti-Vimentin (chicken polyclonal ; Novus Biologicals, Centennial, CO, USA, #NB300-223)<br>Donkey anti-rabbit lgG-HRP (Santacruz, Heidelberg, Germany, SC-2313).<br>Donkey anti-mouse lgG-HRP (Santa Cruz, SC-2314).<br>Goat anti-rat lgG-HRP (Santa Cruz, SC-2020).<br>Goat anti-rabbit AlexaFluor-594 (ThermoFisher, #A11012}.<br>Goat anti-rabbit AlexaFluor-647 (Thermofisher, #A21244).<br>Goat anti-mouse AlexaFluor-568 (Thermofisher,#A11004).
Validation	Anti-β-actin: validated by supplier and supported by citations on supplier website.

Validation

Species Reactivity: Human, Mouse, Rat, Hamster, Monkey, Dog (Species reactivity is determined by testing in at least one approved application (e.g., western blot)).
Applications: Western Blotting, Immunohistochemistry (Paraffin), Immunofluorescence (Immunocytochemistry) and Flow Cytometry.

Anti-KRAP, validated by supplier and supported by citations on supplier website.
Species Reactivity: Human, Mouse, Rat.
Application: WB, IP, IHC, IF,ELISA
Tested Applications: Positive WB detected in rat testis tissue; Positive IP detected in mouse testis tissue.

GFP Tag-Alexafluor-647, validated by supplier and supported by citations on supplier website.
Anti-IP3R1,anti-IP3R2, anti-IP3R3, validated by Taylor lab using cells expressing single IP3R subtypes or no IP3Rs (Fig. 1A in J. Cell Sci. 131, jcs220848).

Anti-STIM1, validated by supplier and supported by citations on supplier website.
Reactivity: Human, Mouse, Rat, Monkey, Bovine (Species reactivity is determined by testing in at least one approved application (e.g., western blot)).
Applications: Immunofluorescence (Immunocytochemistry), Immunoprecipitation, Western Blotting.

Anti-Vimentin, validated by supplier and supported by citations on supplier website.
Reactivity: Human, Mouse, Rat, Porcine, Bovine, Canine, Chicken, Equine
Applications: Western Blot, Immunocytochemistry/Immunofluorescence, Immunohistochemistry, Immunohistochemistry-Frozen, Immunohistochemistry-Paraffin.

Eukaryotic cell lines

Policy information about cell lines

Cell line source(s)

HeLa cells, originally from ATCC.
STIM1-EGFP HeLa cells, generated in this laboratory (PNAS 116, 10392-)
EGFP-IP3R1 HeLa cells, generated in this laboratory (Nat. Commun. 8, 1505-)
HEK cells, Dr David Yule, University of Rochester (to match parentage of HEK-3KO cells)
HEK-3KO (HEK cells in which genes for all 3 IP3R subtypes were disrupted): Kerafast, Boston, MA, USA.

Authentication

Short tandem repeat profiling was used to verify EGFP-IP3R1 HeLa (Eurofins) or HEK-3KO (DNA Diagnostics Center, Fisher Scientific) cells. STIM1-EGFP HeLa cells were not independently authenticated; however, they were generated from the same parental HeLa cell line from which EGFP-IP3R1 HeLa cells were developed. Parental HEK cells were not independently authenticated; however, HEK-3O cells generated from them were authenticated.

Mycoplasma contamination

All cell lines were routinely screened for mycoplasma infection (~3-month intervals throughout the study). None of the lines used were infected.

Commonly misidentified lines
(See ICLAC register)

HEK cells. Although HEK cells were included in the ICLAC register (Registration IDs ICLAC-00063 and ICLAC-00064), we have authenticated the HEK-3KO cell line (generated from the parental HEK-WT cell line) using short tandem repeat profiling. This cell line is particularly useful to study calcium signals in an IP3 receptor-null background.
